# Recent Insights into Long Bone Development: Central Role of Hedgehog Signaling Pathway in Regulating Growth Plate

**DOI:** 10.3390/ijms20235840

**Published:** 2019-11-20

**Authors:** Ryuma Haraguchi, Riko Kitazawa, Yukihiro Kohara, Aoi Ikedo, Yuuki Imai, Sohei Kitazawa

**Affiliations:** 1 Department of Molecular Pathology, Ehime University Graduate School of Medicine, Shitsukawa, Toon City 791-0295, Japan; riko@m.ehime-u.ac.jp (R.K.); kohara.yukihiro.yu@ehime-u.ac.jp (Y.K.); kitazawa@m.ehime-u.ac.jp (S.K.); 2 Department of Diagnostic Pathology, Ehime University Hospital, Shitsukawa, Toon City 791-0295, Japan; 3 Division of Integrative Pathophysiology, Proteo-Science Center, Ehime University Graduate School of Medicine, Shitsukawa, Toon City 791-0295, Japan; aikedo@m.ehime-u.ac.jp (A.I.); y-imai@m.ehime-u.ac.jp (Y.I.)

**Keywords:** hedgehog, growth plate, endochondral ossification, chondrocyte, osteoblast, bone disease

## Abstract

The longitudinal growth of long bone, regulated by an epiphyseal cartilaginous component known as the “growth plate”, is generated by epiphyseal chondrocytes. The growth plate provides a continuous supply of chondrocytes for endochondral ossification, a sequential bone replacement of cartilaginous tissue, and any failure in this process causes a wide range of skeletal disorders. Therefore, the cellular and molecular characteristics of the growth plate are of interest to many researchers. Hedgehog (Hh), well known as a mitogen and morphogen during development, is one of the best known regulatory signals in the developmental regulation of the growth plate. Numerous animal studies have revealed that signaling through the Hh pathway plays multiple roles in regulating the proliferation, differentiation, and maintenance of growth plate chondrocytes throughout the skeletal growth period. Furthermore, over the past few years, a growing body of evidence has emerged demonstrating that a limited number of growth plate chondrocytes transdifferentiate directly into the full osteogenic and multiple mesenchymal lineages during postnatal bone development and reside in the bone marrow until late adulthood. Current studies with the genetic fate mapping approach have shown that the commitment of growth plate chondrocytes into the skeletal lineage occurs under the influence of epiphyseal chondrocyte-derived Hh signals during endochondral bone formation. Here, we discuss the valuable observations on the role of the Hh signaling pathway in the growth plate based on mouse genetic studies, with some emphasis on recent advances.

## 1. Introduction

**Figure 1:**
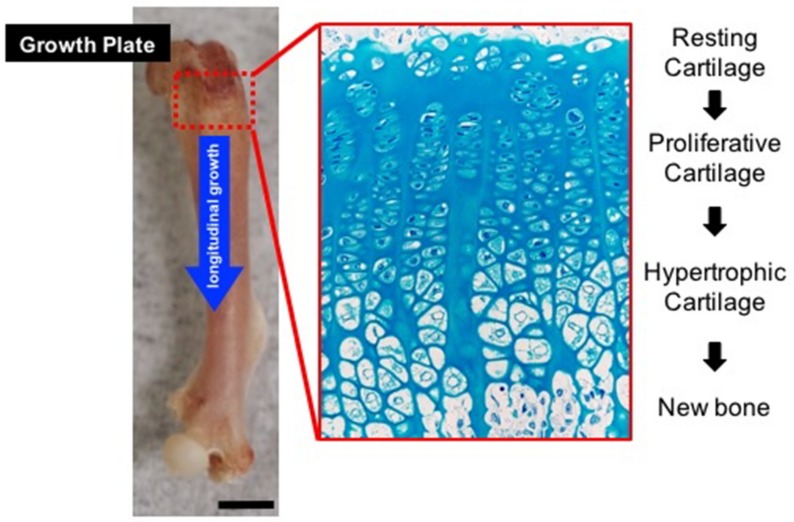
The longitudinal growth of long bone by the growth plate. The growth plate is composed of highly organized and specialized three types of cartilage: the resting, proliferative, and hypertrophic zone. Resting zone chondrocytes supply stem-like cells that give rise to clones of proliferative zone chondrocytes, and determine the spatial orientation of adjacent proliferative columns parallel to the long axis of the bone. The proliferative zone is the region of active cell replication. Hypertrophic zone chondrocytes provide a cartilaginous template, mineralized by their extracellular matrix, supporting the new bone formation by osteoblastic cells. Scale bars indicate 1.25 mm.

The growth plate is a layer of cartilage in developing long bones between the epiphysis and the metaphysis. The elongation of long bones occurs at the growth plate, where cartilage is formed and then replaced by bone tissue (Figure 1). In mammals, the growth plate is composed of three types of highly organized and specialized cartilage: resting, proliferative, and hypertrophic zone, which originate from embryonic cartilage primordia by the condensation of undifferentiated limb bud mesenchymal cells. Resting zone chondrocytes supply stem-like cells that give rise to clones of proliferative zone chondrocytes, and determine the spatial orientation of adjacent proliferative columns parallel to the long axis of the bone [1]. The proliferative zone is the region of active cell replication [2]. When a proliferative zone chondrocyte divides, its derivatives, proliferating rapidly, line up along the long axis of the bone. As a result, clones of chondrocytes are arranged in columns parallel to this axis, and this orientation determines longitudinal bone growth in a specific direction. Proliferative chondrocytes gradually stop dividing and expand to become hypertrophic chondrocytes [3,4]. Hypertrophic zone chondrocytes, terminally differentiated chondrocytes, produce a kind of scaffold, mineralized by their extracellular matrix, that supports bone formation by osteoblastic cells before they undergo apoptosis. Hypertrophic chondrocytes also promote vascular invasion at the chondro-osseous junction (COJ), the junction between calcified and non-calcified cartilage matrices, which is a critical process for recruiting osteoblast and osteoclast progenitors [5,6]. The overall process described above, commonly referred to as “endochondral ossification”, has been studied widely because it regulates the longitudinal growth of bone [7]. Also, a limited number of chondrocytes within the growth plate are themselves generated from stem-cell-like progenitor cells called chondro-progenitors, and directly transform in considerable numbers into the osteogenic lineage in developing bone [8].

**Figure 2:**
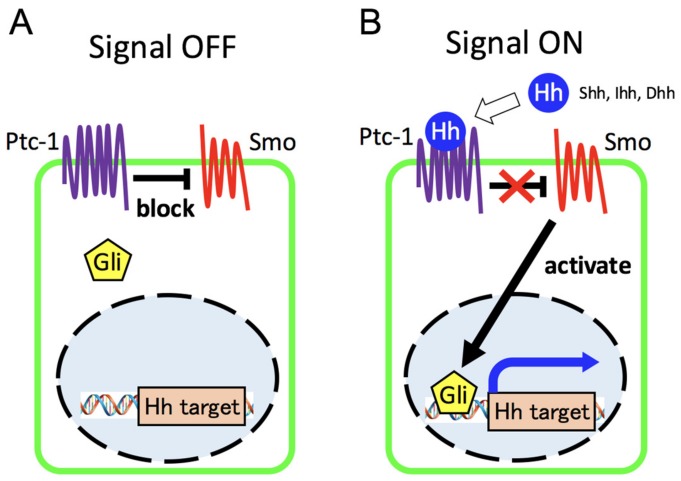
Overview of hedgehog signaling pathway. (**A**) In the absence of Hh ligands, Ptc-1 blocks Hh pathway activation through the repression of Smo. (**B**) Once the Hh ligand binds to Ptc-1, the repressive action to Smo is released, and Gli-mediated transcription leading to the regulation of downstream target Hh is activated.

Hedgehog (Hh) signaling is known to be among the most important regulators in many aspects of insect and vertebrate development [9,10,11,12,13,14,15]. In mammals, the three Hh proteins, Sonic hedgehog (Shh), Indian hedgehog (Ihh), and Desert hedgehog (Dhh), undergo several steps of post-translational modification, including proteolytic cleavage, glycosylation, and lipid modification, after which they are released by Hh-secreting cells with the help of Dispatched (Disp), a membrane transporter protein [10,14,15]. Hedgehog ligands are equally essential during vertebrate embryonic development. Shh is expressed at various tissues, including the brain, skeleton, tooth, skin, gastrointestinal tract, urogenital tract, and lung, Ihh in gastrointestinal tract and cartilage, Dhh in the peripheral nerves and testicular cells [16]. Recently, it has been discovered that Shh also controls the behavior of cells with stem cell properties in the maintenance and regeneration of adult tissues [17]. After post-translational modification Hh proteins transmit signals through a receptor complex that includes the G-protein-coupled receptor, the twelve-pass transmembrane receptor Patched-1 (Ptc-1) and the seven-pass transmembrane protein smoothened (Smo), to control gene expression by modulating the activity of Gli transcription factors (Figure 2) [9,10,11,13,15]. In the absence of Hh ligands, Ptc-1 negatively regulates Hh pathway activation through the constitutive repression of positive Hh effector smoothened and is also transcriptionally controlled. Once the Hh ligand binds to Ptc-1, the repressive action to Smo is released, and Gli-mediated transcription leading to the regulation of downstream target Hh is activated. In mammals, among Gli transcription factors (Gli1/2/3) that collectively mediate all Hh signaling, Gli-2 and Gli-3 are the initial responders to Hh signaling [9,14]. Gli-1 is a positive transcriptional mediator and one of the direct downstream target genes in the Hh pathway. Gli-2 is considered to function predominantly as a transcriptional activator, whereas Gli-3 functions mainly as a repressor (for detailed review, see [9,14,18]).

In general, Ihh participates in the process of endochondral bone formation and is expressed in the hypertrophic zone chondrocytes of the growth plate [19]. Based mainly on in vivo studies with the use of genetically modified mice, remarkable progress has been made in understanding how hedgehog signaling from growth plate chondrocytes regulates skeletal development and interacts with other signaling factors. The multiple functions of Hh signaling in developing long bone have previously been summarized by several research groups [9,15,20]. The present review is aimed at summarizing current findings that would assist in understanding this area of research, with an emphasis on the multiple roles of Hh during early-chondrogenesis and endochondral ossification processes, the coupling function of Hh and cholesterol biosynthesis during chondrocyte differentiation, the potential role of Hh in the cell trans-differentiation of chondrocytes into bone cells, and the involvement of Hh in skeletal disorders.

## 2. Hedgehog Signal Is a Critical Regulator of Early Chondrogenesis

Chondrogenesis is the earliest phase of skeletogenesis that results in the formation of the growth plate and leads to endochondral ossification during the growth of the long bone [21,22,23]. The onset of chondrogenesis is marked by the condensation of dividing undifferentiated mesenchymal cells in the limb bud, and condensed cells subsequently differentiate into clusters of cartilage cells known as chondrocytes that continue to proliferate until their hypertrophic differentiation. This cartilaginous tissue eventually becomes vascularized, initiating the formation of growth plates. Before chondrocyte hypertrophy, the onset of the chondrocyte maturation process, enlargement of the cartilage template by the proliferation of condensed pre-cartilage or cartilage cells, is vital for a well-organized growth plate.

Among the Hh protein family in mammals, Ihh is known to function as a principal source of the Hh ligand that activates the Hh signaling pathway on skeletogenesis, which is primarily expressed in proliferating limb bud mesodermal cells that eventually differentiate into skeletal chondrocytes immediately after mesenchymal condensation [19]. Studies on mice with mutations in the *Ihh* gene have provided in vivo evidence that Hh signaling is requisite for adequate cell proliferation of the condensed pre-cartilage mesenchyme responsible for forming a framework for endochondral ossification [19,24]. Global Ihh knockout mice show a remarkable reduction in longitudinal growth, and most of Ihh-null mutants died at birth, due to respiratory failure [19]. The long bones of Ihh-null mutants are only about one-third the length of those in wild-types. These defects are not directly affected by the chondrocyte maturation process, as is apparent from as early as the mid-embryonic stage prior to cartilage hypertrophy. Moreover, Ptc-1, some of the transmembrane receptor complex for Hh ligands and the direct downstream target of the Hh signaling pathway are expressed at the dividing condensed mesenchyme adjacent to Ihh-expressing cells, and their expression in the Ihh mutant limb is markedly decreased with a significant reduction in the proliferation of cartilaginous tissue [19]. These observations suggest a direct role of the Hh pathway in the cartilaginous growth of limb skeletal elements. In contrast, whereas the Hh pathway is required for limb bud chondrocyte proliferation, its aberrant activation also leads to the dysregulation of chondrogenic skeletal formation. Ligand-independent activation of the Hh pathway has an inhibitory effect on early chondrogenesis [24]. The authors have demonstrated that conditional deletion of the *Ptc-1* gene in the undifferentiated limb mesenchyme with the use of Prx1-Cre, causes cell-autonomously activated Hh signaling cascade, resulting in marked disorganization of skeletal tissues that are severely truncated cartilage elements with a negative Alcian blue staining. Furthermore, an in vitro micro-mass culture system has revealed that activation of ligand-independent Hh signaling prevents early chondrogenesis. Micro-mass cultures derived from Prx1-Cre:Ptc-1^c/c^ limbs show a significant decrease in cartilage cluster formation. Moreover, a decrease in the expression of the *Col2a1* gene, an early chondrogenic marker reflecting the onset of chondrocyte differentiation, is detected in mutant cultures with the upregulation of universal downstream Hh target genes. Under the same experimental conditions, despite an increase in the level of Hh targets, no difference is observed in the expression level of Sox-9, the earliest master regulator of chondrogenesis, or of N-Cadherin, a marker for mesenchymal condensation, in Prx1-Cre:Ptc-1^c/c^ versus the control. These findings of in vitro experiments using the limb micro-mass culture system support the concept that the inhibitory effect of cell-autonomously activated cells of the Hh pathway on early-chondrogenesis underlie below mesenchymal cell condensation and above chondrocyte differentiation. In contrast to the Prx1-Cre:Ptc-1^c/c^ model, exogenous Hh ligand treatment of micro-mass cultures, which is an activation of the ligand-dependent Hh pathway, causes continuous increases in the expression of chondrogenic markers involved in the formation of mature cartilage clusters [24]. Results of Hh ligand treatment on micro-mass cultures is consistent with global Ihh knockout early-stage limb phenotypes. Thus, Hh signaling is most likely related to the rapid enlargement of cartilage tissues during early-chondrogenesis, and this developmental process requires the balancing of positive and negative input involved in the control of the activation level of the Hh pathway.

In addition to the fundamental effector molecules such as *Ptc-1*, *Smo,* and *Gli*, functional genes that directly control Hh signal transduction have been identified by using differential screening and phenotypic analyses of mutant animal models [10,12,13,15]. These genes, including *Kif7*, *Sufu*, *Hhip*, *Cdo*, *Boc,* and *Gas1*, are capable of modulating the activation level of the Hh pathway through direct interaction with Hh ligands and their cytoplasmic components. Functional mutation in these genes exhibits various chondrogenic defects with an alteration of Hh signaling activity during early embryogenesis [25,26,27]. These observations also support the importance of fine-tuned Hh signal activity in early cartilage development, and the mechanisms underlying developmental defects caused by the dysfunction of Hh modifiers need to be elucidated further.

## 3. Central Role of Hedgehog Signaling in Regulation of Growth Plate

In the metaphysis at both ends of a long bone, the growth plate is orchestrated in the limb skeletal cartilage through multistage processes: vascular invasion, the formation of primary/secondary ossification centers and osteoblast/osteoclast recruitment under the influence of regulatory molecules (for detailed review, see [28]). Growth plate chondrocytes undergo a tightly regulated developmental program of proliferation, pre-hypertrophy, hypertrophy, and apoptosis in the specialized cartilage layers and are eventually replaced by osteoblasts at the distal edge of the growth plate (also termed COJ: chondro-osseous junction). The precise regulation of growth plate chondrocytes aligned according to their defined differentiation phase, absolutely crucial for longitudinal growth of endochondral bones, is achieved under the adequately controlled activity of the Hedgehog (Hh) signaling pathway.

### 3.1. Hh Pathway and Growth Plate Formation

In general, among Hh ligands, Indian hedgehog (Ihh) acts in the process of growth plate development [20,28]. As noted above, the *Ihh* gene is initially expressed in condensed limb mesenchymal cells or in chondrocytes of the cartilaginous skeletal elements. During growth plate development, Ihh expression becomes gradually restricted to postmitotic pre-hypertrophic chondrocytes adjacent to proliferative zone chondrocytes.

**Figure 3:**
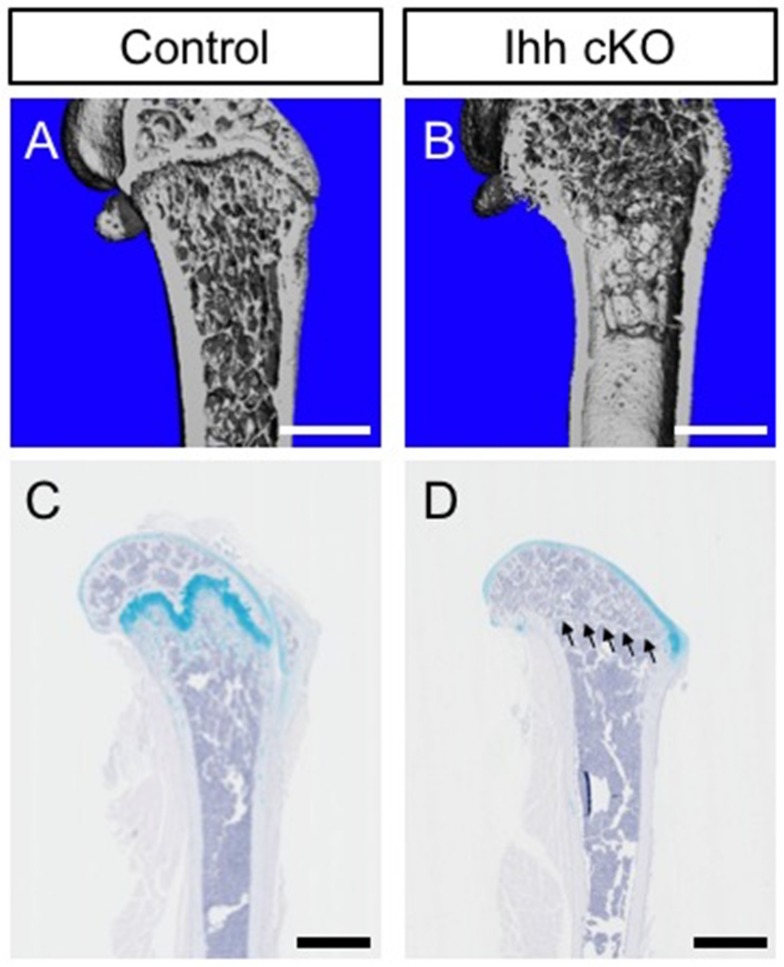
Chondrocyte-derived Ihh is required for the maintenance of a normal growth plate. (**A**,**B**) Longitudinal view of μCT images in distal femur from control and Gli1-CreER; Ihh^c/c^ (Ihh cKO). Control and Ihh cKO littermate mice were treated with tamoxifen at four weeks of age and analyzed after eight weeks to inactivate the *Ihh* gene. Note decreased trabecular mass and completely lacked growth plate in Ihh cKO mice (**B**). (**C**,**D**) Representative images of femur stained with hematoxylin and alcian blue. Alcian blue positive cartilage matrix in the distal femur is absent in Ihh cKO mice (D, arrowheads show). Scale bars indicate 1 mm (**A**,**B**) and 1.25 mm (**C**,**D**).

In vivo studies using Ihh mutant mouse models and our data have revealed that Ihh is indispensable for the process of growth plate organization (Figure 3) [19,29,30,31,32]. These models show abnormal endochondral bone formation with a complete absence of the growth plate and the superiority of mature chondrocytes. Mice carrying null mutations of the *Ihh* gene show a severely disrupted growth plate with abnormal chondrocyte proliferation and maturation at embryonic stages [19]. The conditional ablation of Ihh in the full skeletal lineages of the limb by using *Prx1* promoter markedly inhibits skeletal development in the absence of the normal growth plate and the secondary ossification center in the postnatal period [29,30]. Newborn Prx1-Cre:Ihh^c/c^ growth plate cartilage lacks a zone of aligned columnar chondrocytes and both pre-hypertrophic and hypertrophic chondrocytes are barely formed. Before postnatal day 10, mutant humerus bone revealed a total absence of a growth plate and no secondary ossification center. Ablation of Ihh by using *Col2a1* promoter/enhancer also reveals severe skeletal deformities with loss of a normal growth plate exhibiting the characteristic zones of chondrocyte differentiation (resting, proliferating, pre-hypertrophic and hypertrophic) [32]. The Col2a1-Cre:Ihh^c/c^ disorganized growth plate shows approximately half the number of BrdU-labeling cells and abnormal location (in the central region of the long bone) of hypertrophic cells expressing Type X collagen at late embryonic stages. The mutant growth plate also showed a delay in chondrocyte differentiation, as indicated by the delayed expression of Type X collagen and osteopontin. Moreover, ablation of the *Ihh* gene from postnatal chondrocytes by using tamoxifen-inducible Col2a1-CreER transgenic mouse lines has been shown to cause premature closure of the growth plate: disrupted columnar structure of chondrocytes, and the appearance of abnormal maturation of hypertrophic chondrocytes near the articular surface [31]. The growth plate of neonatal-tamoxifen-injected Col2a1-CreER:Ihh^c/c^ mice has shown complete loss of the columnar structure of proliferating chondrocytes. The mutant growth plate is composed mainly of hypertrophic chondrocytes that express Type X collagen but not Type II collagen, showing an incorrect progressive maturation of cartilaginous cells [31]. This abnormal process that starts at postnatal day seven eventually leads to a total loss of the growth plate in mutant tibial bones at postnatal day 14. Thus, the actions of Ihh in the skeletal cartilage adequately regulate chondrocyte proliferation and maturation required for the organization of the normal growth plate during embryonic and postnatal periods.

### 3.2. Hh Pathway Controls Regulation of Growth Plate Differentiation through Interaction with PTH-PTHrP Signaling

The Hh pathway through Ihh signaling is critical for not only the initial morphogenesis but also the subsequent onset and advancement of chondrocyte differentiation in the growth plate. In vivo genetic studies have verified that the activated Hh pathway controls these processes through interaction with PTH-PTHrP signaling [7,19,20,28,33,34]. Parathyroid hormone-related peptide (PTHrP), which is similar to parathyroid hormone (PTH), plays a crucial role in chondrocyte proliferation and hypertrophy of the growth plate [35,36]. In the growth plate, PTHrP is expressed at high levels in periarticular resting cells and at low levels in proliferating chondrocytes adjacent to the pre-hypertrophic zone, while its receptor, Parathyroid hormone 1 receptor (PTH1R), is produced at low levels by proliferating chondrocytes and at a high level in pre-hypertrophic cells [19,34]. In studies on mice, loss-of- and gain-of-function of PTHrP and PTH1R have indicated that the PTH-PTHrP signal maintains chondrocytes proliferating in the growth plate and suppresses their excessive hypertrophy, resulting in premature mineralization of growth plate chondrocytes [37,38]. Basic studies on animal models have demonstrated that PTHrP production in the growth plate is controlled by the Hh pathway through Ihh signaling. As noted above, Ihh is expressed and secreted by pre-hypertrophic and hypertrophic chondrocytes in the growth plate, while PTHrP is expressed in periarticular resting cells and proliferating chondrocytes adjacent to the Ihh expressed pre-hypertrophic zone. A study on a chicken embryo model has demonstrated that overexpression of Ihh increases PTHrP expression in periarticular chondrocytes in the growth plate [34]. Hh pathway may not directly control the promoter activity of PTHrP [39,40]. PTHrP expression is absent from the growth plate in Ihh-null mice that exhibit a skeletal phenotype (leading to accelerated hypertrophy of chondrocytes) similar to that caused by the *PTHrP* gene deletion [19]. Studies with the use of compound mutant mice have demonstrated that constitutive activation of the PTHrP signal in the Ihh-null growth plate partially rescues its abnormality. Double mutant growth plates do not accelerate chondrocyte hypertrophy, suggesting that the Hh pathway (through Ihh) controls growth plate development by a PTHrP-dependent pathway [41,42]. PTHrP regulated by Ihh probably plays a critical role in fine-tuning between chondrocyte proliferation and maturation.

Ihh promotes chondrocyte differentiation hypertrophic chondrocytes; at the same time, PTHrP expression induced by Ihh maintains the proliferating state of chondrocytes and blocks their hypertrophic differentiation [7,20,28]. Maintenance of the proliferation state in the growth plate eventually delays Ihh production by hypertrophic chondrocytes. Thus, Ihh and PTHrP form a negative feedback loop that both synchronizes and controls the ratio of chondrocyte proliferation and differentiation in the growth plate.

### 3.3. Crosstalk between Hh and Other Signaling Pathways as a Basis for Regulatory Mechanisms of Growth Plate Development and Function

In vivo studies based on genetic manipulation of mice strongly suggest the possibility of signaling crosstalk underlying strict regulation of growth plate development and function by Hh pathways and other signaling pathways [20,28,43,44].

#### 3.3.1. Hh and Wnt/β-Catenin Signaling

Wnt/β-catenin signaling that regulates osteoblast maturation could be affected by the functional deletion of Ihh from postnatal chondrocytes. It has been identified as playing fundamental roles in growth plate formation and in the terminal differentiation of osteoblasts from their progenitors adjacent to the growth plate [31].

In neonatal-tamoxifen-injected Col2a1-CreER:Ihh^c/c^ mice, efficient deletion of the *Ihh* gene from postnatal growth plate chondrocytes has shown a significant reduction in β-catenin expression in the bone collar and primary trabeculae of mutants. Moreover, a remarkable reduction in the expression of Dickkopf1 (Dkk1) and osteoprotegerin (OPG), the downstream target gene of the Wnt/β-catenin signaling pathway, has been evident in the mutants [31]. Furthermore, compound mutant analysis has shown that the Wnt/β-catenin signaling pathway is a critical downstream target of Hh signaling from chondrocytes for the regulation of osteoblast differentiation during endochondral ossification. Also, Hh signaling is activated and Wnt/β-catenin is inactivated by a chondrocyte-specific deletion of Ptc-1 and β-catenin in mice treated with *Col2a1* promoter/enhancer [45]. By expression analyses of Hh signaling target genes, Hhip and Gli-1 marked activation has been found in both Ptc-1 mutant and double mutant mice in terms of Ptc-1 and β-catenin, which indicates that Wnt/β-catenin is requisite for bone formation and acts downstream of the Hh pathway. Thus, these observations support the view that growth plate chondrocyte-derived Ihh is critical for skeletal formation through the activation of the Wnt/β-catenin pathway and for regulating its action.

#### 3.3.2. Hh and FGF Signaling

Fibroblast growth factor (FGF) signaling has been identified as playing fundamental roles in the proliferation and differentiation process of growth plate chondrocytes [46]. The FGF family comprises at least 22 ligands that bind to at least four receptors, among which FGF receptor-3 (Fgfr-3) critically regulates endochondral bone formation in the growth plate [47,48]. Mice carrying null mutations of Fgfr-3 display accelerated long bone elongation, a high rate of chondrocyte proliferation and enlargement of chondrocyte columns in the hypertrophic zone [49,50]. Conversely, the gain-of-function mutation of Fgfr-3 reduces chondrocyte proliferation and results in a markedly shortened long bone with disorganized chondrocyte columns [51,52,53,54,55]. Minina et al. have shown that the inhibition of growth plate chondrocyte proliferation, by upregulated FGF signaling through Fgfr-3 activation, is caused partly through the inactivation of the Hh pathway through Ihh signaling [56]. They have observed that dominantly activated Fgfr-3 reduces Ihh expression in hypertrophic chondrocytes of the growth plate. In vitro studies using the limb culture system have shown similar results, indicating an antagonistic action of FGF signaling in the control of chondrocyte proliferation and in Ihh expression [56]. Also, the Hh signaling pathway is dysregulated in the Fgfr-3 mutant growth plate [57]. As noted above, normally Ihh is expressed in growth plate chondrocytes of pre-hypertrophic and hypertrophic zones. By contrast, Fgfr-3-deficient mice show a markedly upregulated Ihh expression in the disorganized mutant growth plate, revealing an increase in proliferating chondrocytes and expansion of the hypertrophic zone. Moreover, systemic inhibition of the Hh pathway by smoothened inhibitor treatment partially prevents growth plate defects of Fgfr-3 mutants [57]. Thus, FGF signaling through Fgfr-3 activation controls the balance between proliferation and maturation of growth plate chondrocytes by fine-tuning the Hh pathway and suppressing Ihh expression.

#### 3.3.3. Hh and BMP Signaling

Bone morphogenetic protein (BMP) signaling plays a vital role in endochondral bone development [7,28,58,59,60]. In vitro studies have shown that the addition of BMPs to limb explant culture systems enhances chondrocyte proliferation, and their effect is blocked by noggin, an inhibitor of several BMPs; also, that Bmp-2 stimulation delays terminal differentiation of hypertrophic chondrocytes [61]. Presently, various in vivo studies on loss-of-function have confirmed these actions of BMP signaling. Bmp-2 conditional deletion mice using the Col2a1-CreER line have demonstrated the failure of chondrocyte proliferation and maturation in the growth plate [62]. Chondrocyte specific deletion mice of BMP signal core receptors, BMPR-IA expressed throughout the growth plate, have demonstrated a disorganized epiphysis and absence of the growth plate [59,63]. Also, conditional deletion of Smad proteins (Smad1 and Smad5), main signal transducers for BMPR, leads to severe malformed growth plates and impaired chondrocyte survival [64]. Previous reports have described the interaction of Hh and BMP signaling in endochondral bone development [61,64]. Studies with the use of the limb explant culture system have demonstrated that BMP and Ihh signaling interact to coordinate chondrocyte proliferation and differentiation, for example, Bmp-2 treated limb explants show increased Ihh expression by hypertrophic differentiation, and promote both the proliferation of chondrocytes and the elongation of proliferative chondrocyte columns. Moreover, enhanced Ihh signaling by *Col2* promoter transgenic model delays hypertrophic differentiation with the upregulation of *Bmp* genes [61]. Furthermore, the canonical Smad pathway triggered by BMPs actually acts as an upstream regulator of Ihh/PTHrP signaling in the growth plate. Chondrocyte specific Smad1/5 conditional KO mice demonstrate severe chondrodysplasia (as mentioned above). The expression of Ihh and PTHrP receptors is completely lacking in the mutant growth plate [64]. Studies have shown that Ihh is a target of BMP and FGF pathways in chondrocytes and that Smad proteins can bind to the *Ihh* promoter region [65]. Interestingly, Smad1/5 cKO analyses have revealed an imbalance of BMPs and FGF signaling in mutant cartilage: disrupted expression of BMP signaling components and advanced phosphorylation and nuclear entry of STAT1, one of the core mediators in FGF signaling [64]. Furthermore, characterization and functional analysis of the promoter of *Ihh* and *Msx2*, one of the downstream targets in BMP signaling, under the influence of BMP and FGF signaling, has disclosed that BMP signaling controls the Ihh/PTHrP signaling loop by inhibiting the antagonistic effect of FGFs on Ihh signaling (as noted above). From these results, it has been proposed that the enhancement of Ihh expression triggered by BMP signaling might be negatively controlled by FGF activation through BMP canonical Smad phosphorylation [64]. Taken together, these observations suggest that BMP signaling has two established functions by cooperating with Ihh (or FGF) signaling in growth plate development: 1) activation of chondrocytes within the resting zone to enter a proliferative state, and 2) prevention of chondrocyte hypertrophy.

#### 3.3.4. Hh Signaling and Angiogenic Factors

At the chondro-osseous junction (COJ), the onset of the final step of chondrocyte hypertrophy is initiated by loss of hypertrophic marker genes, tightly synchronized with the induction of vascular endothelial growth factors (VEGFs) and metalloproteinases (MMPs) [66]. VEGF signaling plays critical roles in promoting vascular invasion and consequent remodeling of cartilage matrices by recruiting osteoblast and osteoclast progenitors. Systemic inhibition of VEGFs by the administration of its soluble receptor suppresses blood vessel invasion and metaphyseal bone trabeculae formation with an increased width of the hypertrophic zone of the growth plate [5]. Mmp-9 and Mmp-13 are prerequisites to the promotion of vascular invasion into the non-calcified hypertrophic matrix (the lacunae of dying hypertrophs, which do have a mineralized ECM [67]): compound KO mice with deleting both genes show marked enlargement in the hypertrophic zone of the growth plate [68]. Previous studies have demonstrated that Ihh signaling by hypertrophic chondrocytes plays critical roles in orchestrating the above vascular invasion and bone remodeling processes at COJ [31,69]. Ihh-null mutant mice display a significant decrease of VEGF-A, Mmp-9 and Mmp-13 in the disorganized growth plate, with no osteoclast staining positive for a TRAP [69]. Chondrocyte specific Ihh-deficient mice generated by using *Col2a1* promoter/enhancer also show atypical vascular invasion in the central region of the mutant growth plate [31]. It has also been suggested that Ihh indirectly regulates VEGF expression of hypertrophic chondrocytes through Runx2 [70,71]. Runx2, a member of the runt family of transcription factors, plays critical roles in the maturation processes of growth plate chondrocytes under the influence of Ihh signaling [70]. Furthermore, Runx2 plays a role in the induction of VEGF expression in hypertrophic chondrocytes of the growth plate [71]. Thus, these observations strongly suggest the existence of a certain link between Ihh signaling and terminal phase regulation of chondrocyte hypertrophy, which includes vascular invasion and cartilage matrix remodeling.

As shown above, this chapter described crosstalk between the Hh pathway and other signaling factors, Wnt/β-catenin, FGFs, BMPs, and VEGF, which are considered essential for normal growth plate development. In addition to the above, however, numerous other signaling pathways contribute to the regulation of growth plate development, and the relation between the Hh pathway and those pathways has yet to be exhaustively elucidated. We believe that the identification of regulatory signaling interaction with the Hh pathway may reveal additional fundamental molecular mechanisms, like Ihh/PTHrP signaling, that dominate growth plate development.

### 3.4. Coupling Role of Hh Signaling Pathway and Intracellular Cholesterol Production in Growth Plate Development

Dysregulation of cholesterol synthesis is involved in multiple developmental abnormalities. Numerous human mutational and clinical studies provide the notion that cholesterol is vital for normal skeletal development [72]. Also, previous experimental studies, on animal models treated with cholesterol synthesis inhibitors, have demonstrated severe skeletal malformations, including digit patterning defects, and decreased width of the long bone growth plate [73,74]. In mammals, cholesterol is produced from steroid hormones, bile acids, and vitamins, and intracellular cholesterol biosynthesis is tightly controlled by proteins in the endoplasmic reticulum (ER), including sterol regulatory element-binding proteins (SREBPs) and SREBP cleavage-activating proteins (SCAP) [75]. *SREBP* genes are activated in response to low cellular cholesterol levels by events of protease cleavage and transport into the nucleus. SCAP constitutes a complex with SREBP and acts as a cholesterol sensor. In the case of low cholesterol levels, SCAP recruits SREBP to the Golgi where proteases cleave SREBP, thereby releasing the N-terminal active domain of SREBP into the nucleus. More recently, studies with genetically modified mice have revealed that adequate regulation of cellular cholesterol biosynthesis in the growth plate chondrocyte is requisite for normal endochondral ossification and maintenance of chondrocyte homeostasis [76]. SCAP conditional deletion mice using the Col2a1-Cre line have displayed disordered growth plates and severe dwarfism. The mutant growth plate has displayed abnormal primary ossification, disorganized round cells in the resting zone, disrupted columnar structures in the proliferation zone, and reduction in the hypertrophic zone.

Although it has been strongly suggested that cell-autonomous cellular cholesterol production is critical for the organization of the normal growth plate, it is additionally represented that the Hh pathway and cellular cholesterol biosynthesis regulate each other during growth plate formation [76]. As mentioned above, the Hh pathway is involved in the control of chondrocyte differentiation in growth plate development, as a fundamental regulatory signal. Previous work has shown that Hh signaling regulates genes encoding intracellular cholesterol biosynthesis in chondrocytes [77]. In the *Col2* promoter transgenic model, enhanced Hh signaling by the overexpression of Gli-2 using *Col2* promoter induces higher levels of cholesterol and lipid accumulation in chondrocytes. By contrast, cholesterol is also capable of controlling Hh signaling at multiple phases in its signaling process, from ligand processing to coordination of receptors and intracellular effectors. Cholesterol modification of Hh ligands is needed for the construction of soluble multimeric Hh protein complexes that are freely diffusible, accumulate in a gradient, and are able to directly activate signaling over long distances [78]. Cholesterol also activates membrane protein smoothened by binding to its extracellular domain [79]. Furthermore, a study has indicated that chondrocyte-specific ablation of SCAP leads to the reduction of the type X collagen positive hypertrophic zone with decreasing expression of Ihh and Hh target genes, and exogenous cholesterol treatment slightly reinstates the reduction of Hh target gene expression in Scap-deficient chondrocytes. Also, enhanced activation of the Hh pathway by Gli-2 overexpression partially rescues the truncated limb phenotype of SCAP deficient mice [76]. Thus, these observations suggest that cholesterol biosynthesis is controlled by the Hh pathway, which is, in turn, controlled by intracellular cholesterol levels in chondrocytes. Detailed analyses of this relationship need to be prioritized in future studies on long bone development.

### 3.5. Hh Pathway and Developmental Contribution of Growth Plate Chondrocytes to Skeletal Bone Formation

Longitudinal bone growth progresses by continuous bone replacement of the growth plate, which is organized into distinct zones of chondrocytes: resting, proliferative, pre-hypertrophic, and hypertrophic. During longitudinal bone growth throughout postnatal and juvenile periods until early adulthood, chondrocytes of the growth plate continue to produce new cartilage matrices that are replaced by bone at the chondro-osseous junction (COJ). Subsequently, chondrocytes at the edge of the developing hypertrophic zone largely disappear by apoptosis as the cartilage matrix is degraded, a process concurrent with the invasion of blood vessels, hematopoietic cells, and progenitors for osteoblasts and marrow adipocytes. Nonetheless, in contrast with the above canonical pathway of endochondral bone formation, there is now a new emerging concept: direct trans-differentiation (chondrocyte-to-osteoblast) of growth plate chondrocytes into bone cells during longitudinal bone growth [8]. This concept is supported by recent genetic lineage tracing studies of growth plate chondrocytes, using constitutively active and inducible Cre-based transgenic mice, such as Acan-Cre-, Col2-Cre-, Col10-Cre- and Sox9-Cre-lines [80,81,82,83]. These studies have demonstrated that reporter gene expressing cells derived from growth plate chondrocytes are detected in the osteoblasts and osteocytes of trabecular and cortical bone, and in the bone marrow stroma during longitudinal bone growth. These lineage tracing experiments have also revealed that early-postnatal labeled chondrocytes in the growth plate contribute to multiple skeletal lineages and continue to supply these progeny cells for the long-span, over a year. Thus, growth plate chondrocytes provide opportunities for controlling skeletal formation that occurs rapidly and uniquely in longitudinally growing bone.

**Figure 4:**
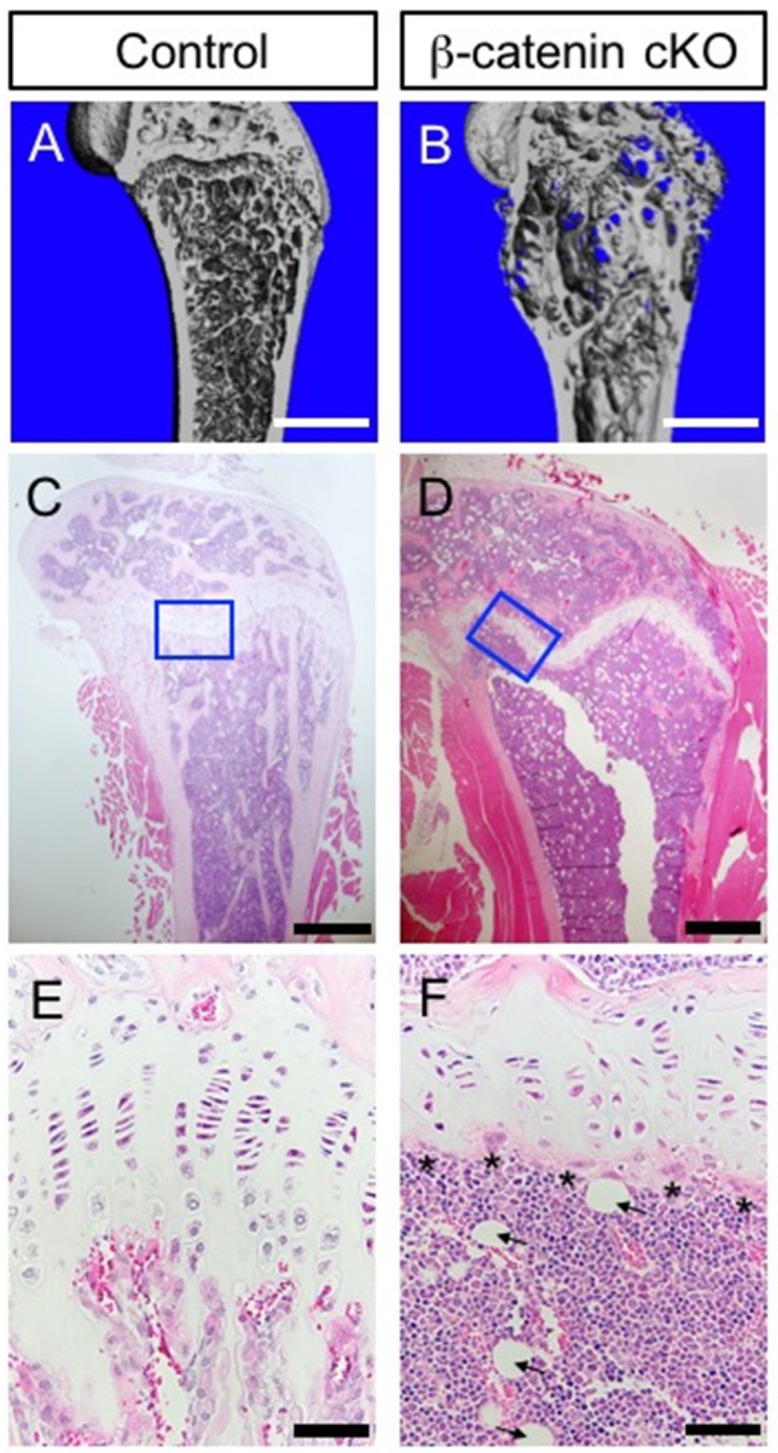
Loss of *β-catenin* gene in growth plate derived Hh-signal responded cells results in osteopenia and fatty bone marrow. (**A**,**B**) Longitudinal view of μCT images in distal femur from control and Gli1-CreER; β-catenin^c/c^ (β-catenin cKO). Control and β-catenin cKO littermate mice were treated with tamoxifen at 4 weeks of age and analyzed after 10 weeks to inactivate the *β-catenin* gene. μCT imaging revealed that β-catenin deletion resulted in an abnormal bone formation in the distal femur. (**C**–**F**) Representative images of femur stained with hematoxylin and eosin. (**E**,**F**) Higher magnification of blue boxes in (**C**,**D**). Histology revealed a lack of trabecular bone under the abnormal growth plate of β-catenin cKO mice (**D**,**F**). This bone phenotype was likely due to increased osteoclastic bone resorption (F, Asterisks mark increased osteoclasts). Histology of the femur also indicated a significant increase in adipocytes at the metaphysis (F, arrowheads show). Scale bars indicate 1 mm (**A**,**B**), 500 μm (**C**,**D**) and 50 μm (**E**,**F**).

More recently, genetic lineage tracing analyses focusing on the Hh pathway have provided evidence that the contribution of growth plate chondrocytes to skeletal lineage formation is regulated under the influence of Hh responsiveness in growing long bone [84,85]. As mentioned above, the Hh pathway through Ihh signaling by hypertrophic chondrocytes has been shown as a critical factor for adequate differentiation of immature growth plate chondrocytes into a hypertrophic state through crosstalk with various signaling pathways. Fate mapping studies by using the Gli1-CreER line, in which the endogenous *Gli-1* gene (one of the Hh pathways downstream of target genes) promoter contains Cre recombinase, have demonstrated that Gli1-CreER genetically labeled cells are observed in hypertrophic chondrocytes and osteoprogenitors at the chondro-osseous junction (COJ). Genetically labeled osteoprogenitors then commit to the osteogenic lineage in the periosteum, trabecular, and cortical bone along the developing longitudinal axis, and continue to supply these progenitor cells for over a year. Our data and studies by others support the concept that correctly regulated Hh-signal responsive cells within the growth plate are functionally crucial for maintaining skeletal bone formation during postnatal life (Figure 3 and Figure 4) [85]. Furthermore, these studies have shown that in aged bone, where longitudinal bone growth ceases, Hh-signal responsiveness and its implication in osteogenic lineage commitment is markedly reduced [84,85]. This observation affirms that age-related regulation of Hh-responsiveness in the growth plate may be one of the key regulatory factors that affect cessation of longitudinal bone growth with age.

The major finding from the above studies (Haraguchi et al. [84] and Shi et al. [85]) is that Hh-responsive cells in the growth plate comprise osteogenic progenitors that can differentiate into osteoblast directly. Congenital and traumatic defects of the growth plate produce a wide range of skeletal disorders including growth retardation, fragmentation, and degeneration with resultant abnormalities of growth [86,87]. The dysregulation of the Hh or other signaling pathways resulting in a permanent anomaly of the growth plate-derived osteogenic lineage is one of the causative mechanisms of skeletal dysplasia in humans. Further understanding of the molecular regulatory mechanism of growth plate chondrocytes transitioning to the growing long bone may help to improve the treatment of skeletal growth disorders.

## 4. Aberrant Hedgehog Signaling in Skeletal Disease

The Hh pathway entails a complicated sequence of regulatory events, including the production and spread of the mature Hh from ligand-secreting cells, tuning of Hh-signal responsiveness in ligands receiving cells, and intercellular coordination of Hh signal transduction activity. Abnormalities of the Hh pathway in the above events cause various bone diseases. As shown with evidence from animal studies, several other reports have shown that Hh signaling regulates and is requisite for bone development and growth in humans.

### 4.1. Hedgehog Signalling and Brachydactyly Syndrome

Brachydactylies are one group of congenital skeletal abnormalities that feature mainly truncated phalanges and/or metacarpals [88,89,90]. Mutational analyses have indicated that three heterozygous missense mutations in IHH cause brachydactyly type A1 (BDA1; OMIM 112500), which features truncated or lacking phalanges [88]. Analyses of Ihh deficient mice have defined the relation between IHH mutations and BDA1 as disturbed Hh pathway through Ihh signaling leading to truncated limbs [91]. Mutations responsible for BDA1 have been restricted to the N-terminal domain of IHH, and for the most part have altered codon positions 95, 100, and 131 [92,93,94]. The DBA1 mouse model, generated with the use of Ihh point mutated mice, had one of the mutations, E95K, inserted into the mouse *Ihh* gene locus; the result was that the point mutated mice demonstrated shortened middle phalanges in digits II and V [91]. Thus, the BDA1 mutation (E95K) results in an alteration of the signaling range and binding capacity of the IHH protein in the interaction with Hh co-receptors, such as PTC-1 and antagonist HHIP. Structural analyses have revealed features of the mutations that cause BDA: 1) E95K mutation is involved in the morphogenetic gradient of the IHH protein in vivo, 2) E95K and D100E mutations result in instability of the N-terminal domain of IHH (IHH-N) with enhanced intracellular degradation at the lysosome, 3) E95K and E131EK mutations affect multimeric formation and cholesterol modification of IHH-N, 4) all three mutations affect the binding capacity of IHH-N to the receptor PTC-1 [93]. These observations imply that Hh mutations impair interaction with Hh receptors and strongly implicate changed Hh signaling capacity and range in the pathogenesis of brachydactyly.

### 4.2. Hedgehog Signaling and Cartilage Tumorigenesis

Cartilaginous tumors are the most frequently occurring benign neoplasms in the skeleton [95,96,97]. The common lesions, enchondroma and osteochondroma, that form adjacent to growth plates during skeletal development, have the potential for malignant change to chondrosarcoma. Cartilaginous tumors arise as a result of mutations in several genes [98,99,100,101,102,103]. Patients with enchondromatosis (Ollier disease and Maffucci syndrome, OMIM 166000) are endowed with inactivating mutations in the Parathyroid hormone 1 receptor (PTH1R), while mice with the PTH1R mutation at codon 150 develop multiple enchondroma-like lesions with upregulated Hh signaling [100,104]. Hereditary multiple exostoses syndrome (HME; OMIM 133700) is associated with heterozygous mutations in *EXT* genes (*EXT1* and *EXT2*), which encode glycosyltransferases that catalyze the polymerization of heparan sulphate (HS) chains [101,102]. Ext1 or Ext2 deleted cells do not synthesize sufficient amounts of HS-rich proteoglycan (HSPG), which is vital for the regulation of the binding and diffusion of Hh ligands on the cell surface [105,106]. Although Ext1/2 KO mice develop skeletal lesions similar to osteochondroma in HME with an abnormal extracellular distribution of Hh ligands [107], recent studies have demonstrated that the autosomal dominant disorder metachondromatosis (MC; OMIM 156250), a rare disease characterized by enchodroma and osteochondroma, is found to be involved in heterozygous loss-of-function mutations in tyrosine-protein phosphatase non-receptor type 11 (PTPN11), encoding protein tyrosine phosphatase SHP2 that relays signals from the activated Ras/extracellular signal-regulated kinase (ERK) pathway [98,103]. Analysis of Ptpn11-deficient mice has revealed the association between PTPN11 mutations and MC, just as the inactivated Ptpn11 pathway in KO mice leads to lesions very similar to MC, and mutant chondroprogenitors enhances Ihh expression. Interestingly, in all the above syndromes showing cartilaginous tumors, aberrant activation of the Hh pathway is observed in their cartilaginous lesions [108]. These findings strongly support the view that over-activated Hh signaling at the growth plate is sufficient to cause cartilaginous neoplasms and that some regulatory signaling including PTH/PTHrP, EXTs, and PTPN11 acts as a tumor suppressor in cartilaginous tissues through the inhibition of Hh signaling.

### 4.3. Hedgehog Signaling and Heterotopic Ossification

Progressive osseous heteroplasia (POH; OMIM 166350) is an autosomal dominant skeletal disorder characterized by widespread heterotopic ossification of skeletal muscle and deep soft connective tissue [109,110]. POH has been described as caused by loss-of-function mutation of GNAS encoding the stimulatory alpha subunit, Gαs, that transduces signals from G protein-coupled receptors (GPCRs) [111,112]. The main phenotypical indication of POH is advanced articular deformation and growth retardation, which are caused by ectopic ossification from embryonic mesenchymal progenitor cells. Analysis of Gnas-deficient mice has revealed the underlying molecular mechanism of POH pathogenesis. Mice carrying tissue-specific mutations of Gnas using Prx1-Cre transgenic driver line display POH-like skeletal anomalies with ectopic expression of osteogenic markers, and aberrant mineralization disclosed by Von Kossa staining [112]. Interestingly, in Gnas-deficient cells, the Hh pathway is activated as indicated by the higher expression of Hh target genes, Ptc-1, Gli-1, and Hhip, and Hh signaling is upregulated in patients with POH. Furthermore, another analysis has also demonstrated that Gnas acts through cAMP and PKA, downstream pathways of Gnas, to suppress Hh signaling and that reducing Hh signaling activity partially improves the phenotypes of POH [112]. These findings have provided strong evidence that Hh signaling is closely associated with Gnas in skeletal development. In soft tissues without ossification, such as muscle and skin, the activity of the Hh pathway may be rigorously regulated by the GPCR pathway through GNAS to prevent ectopic bone formation during early skeletal genesis.

Abundant genetic evidence, that the Hh pathway plays a central role during skeletal formation, has been accumulated over the past two decades, and ongoing studies for the integrated understanding of its dysregulation and development in human skeletal disorders continue to the present day. A great number of researchers and clinicians suggest that the Hh pathway represents a novel drug target with therapeutic potential in diseases, and some pharmacological materials that adjust Hh signaling activity are being utilized annually. Blocking the Hh pathway may help to improve the treatment of heterotopic ossification, or cartilaginous tumors. Conversely, activation of Hh signaling may be effective in the promotion of osteogenesis for tissue repair and recovery from skeletal deformities, or traumas. Thus, maintaining adequate Hh signaling activity can be thought of as a key element for sustaining healthy skeletal homeostasis.

## 5. Concluding Remarks

In this paper, we have reviewed the multiple roles of the Hh pathway in the regulation of growth plate formation and differentiation. During early chondrogenesis, the Hh pathway promotes cartilaginous growth in condensed limb mesenchymal cells. After organizing the growth plate, the Hh pathway, through Ihh signaling by hypertrophic chondrocytes, regulates chondrocyte differentiation by interacting with PTH-PTHrP signaling, which is termed the PTHrP-Ihh feedback loop system. Other regulatory pathways, such as Wnt/β-catenin, FGFs, BMPs, and VEGF, also interact with the Hh pathway in regulating the growth plate. Moreover, given that the Hh pathway and cellular cholesterol biosynthesis regulate each other during growth plate formation, Hh may be associated with bone diseases related to steroid hormones. Furthermore, recent fate-mapping studies have provided particular evidence showing that epiphyseal hypertrophic chondrocytes under the influence of Hh signaling include osteogenic progenitors that can differentiate into the skeletal lineage for longitudinally growing bone.

The action of the Hh signal in developing long bone is one of the most promising paradigms for understanding the key developmental mechanisms controlled by a growth plate. Future studies are needed to define the precise developmental role of signaling cascades, which is important for understanding skeletal formation (Fgf, Wnt, Bmp, etc.) within Hh-signal-responsive cell lineages originating from the growth plate. Elucidation focused on the regulatory mechanisms of growth plate by Hh pathway would have a positive impact on the full understanding of longitudinal bone development and skeletal disorders.
